# New Insights into the Pathogenesis of Systemic Mastocytosis

**DOI:** 10.3390/ijms22094900

**Published:** 2021-05-05

**Authors:** Zhixiong Li

**Affiliations:** Department of Hematology, Hemostasis, Oncology and Stem Cell Transplantation, Hannover Medical School, 30625 Hannover, Germany; li.zhixiong@mh-hannover.de; Tel.: +49-511-532-9383; Fax: +49-511-532-18586

**Keywords:** systemic mastocytosis, *KIT* D816V mutation, pathogenesis, targeted therapy, *TRK*

## Abstract

Mastocytosis is a type of myeloid neoplasm characterized by the clonal, neoplastic proliferation of morphologically and immunophenotypically abnormal mast cells that infiltrate one or more organ systems. Systemic mastocytosis (SM) is a more aggressive variant of mastocytosis with extracutaneous involvement, which might be associated with multi-organ dysfunction or failure and shortened survival. Over 80% of patients with SM carry the *KIT* D816V mutation. However, the *KIT* D816V mutation serves as a weak oncogene and appears to be a late event in the pathogenesis of mastocytosis. The management of SM is highly individualized and was largely palliative for patients without a targeted form of therapy in past decades. Targeted therapy with midostaurin, a multiple kinase inhibitor that inhibits KIT, has demonstrated efficacy in patients with advanced SM. This led to the recent approval of midostaurin by the United States Food and Drug Administration and European Medicines Agency. However, the overall survival of patients treated with midostaurin remains unsatisfactory. The identification of genetic and epigenetic alterations and understanding their interactions and the molecular mechanisms involved in mastocytosis is necessary to develop rationally targeted therapeutic strategies. This review briefly summarizes recent developments in the understanding of SM pathogenesis and potential treatment strategies for patients with SM.

## 1. Introduction

Mastocytosis is a myeloid neoplasm characterized by clonal expansion and the accumulation of morphologically and immunophenotypically abnormal mast cells (MCs) in one or more organ systems [1,2]. Mastocytosis patients show heterogeneous clinical presentations. The skin-limited presentation, cutaneous mastocytosis (CM), is particularly common among pediatric patients, with disease onset most frequently occurring within the first 2 years of life and commonly experiencing spontaneous regression of skin lesions, In contrast to CM, systemic mastocytosis (SM) associated with extracutaneous involvement is generally observed in adult patients. SM is generally more aggressive and may be associated with multi-organ dysfunction or failure and shortened survival [3,4,5,6]. The World Health Organization (WHO) classification of SM has been demonstrated to be prognostically relevant. The WHO classification is a useful first step for establishing prognosis, based on the following categories: indolent SM (ISM), aggressive SM (ASM), SM associated with a clonal hematological non-MC lineage disease (SM-AHNMD, now known as SM with an associated hematological neoplasm [SM-AHN] per the 2016 WHO classification guidelines [7]), and mast cell leukemia (MCL) [3,5]. Advanced SM (ASM, SM-AHN, and MCL) is generally associated with a poor prognosis, whereas ISM patients usually have a comparable life expectancy as the general population.

The molecular mechanisms that underlie SM development are not well-understood. Over 80% of patients with SM harbor the *KIT* D816V mutation [1]. However, *KIT* D816V mutation is a weak oncogene and appears to be a late event in the pathogenesis of mastocytosis [8]. Although KIT inhibitors present good inhibitory effects against MCs in vitro, treatment with KIT inhibitors alone has been disappointing in most published clinical trials for mastocytosis [1,4,9], likely due to the development of resistance against kinase inhibitors. The management of SM is typically highly individualized and was largely palliative for patients without a targeted form of therapy in past decades [10]. Recently, the US Food and Drug Administration (US FDA) and the European Medicines Agency (EMA) approved midostaurin (PKC412), a multiple kinase inhibitor that also inhibits KIT, for the treatment of advanced SM. Targeted therapy with midostaurin has shown efficacy in patients with advanced SM; however, overall survival in SM remains unsatisfactory, with a 3-year survival of 46% [11,12]. The new KIT inhibitor Avapritinib (BLU-285) also yielded very promising results in a recent study [13], but only a few patients (2 of 18) experienced a complete response. Allogeneic hematopoietic stem cell transplantation may represent a viable and potentially curative therapeutic option for advanced SM, although its definitive role as a treatment strategy remains to be determined [4]. Allogenic natural killer (NK) cells can efficiently eradicate myeloblasts but not malignant MCs in SM associated with acute myeloid leukemia (AML) [14]. Taken together, these findings underscore the need to better understand SM pathogenesis for the development of more efficient treatment strategies. New developments, including the use of next-generation sequencing (NGS) panels and the increasingly sensitive detection of the KIT D816V mutation have improved our understanding of SM pathogenesis. In this review, I discuss the important somatic molecular events that occur during the development of SM.

## 2. Molecular Events in the Development of SM

### 2.1. KIT

The interaction between KIT and its ligand, stem cell factor (SCF), plays an essential role in the regulation of MC maturation, proliferation, adhesion, chemotaxis, and survival [1,15,16]. Gain-of-function somatic mutations in the KIT tyrosine kinase domain, particularly the D816V mutation, have been identified in most adult SM patients, irrespective of the WHO SM subtype. Other, less common (<5%) somatic *KIT* mutations have also been identified in adult SM patients, including V560G, D815K, D816Y, D816F, D816H, and D820G. Interestingly, the *KIT* D816V mutation was detected in 84% of men and 75% of women analyzed (*p* < 0.001), which is consistent with generally worse outcomes observed for male patients with SM [17]. Generally, the *KIT* D816V mutation is less frequently detected in cases of childhood-onset mastocytosis than in adult patients (approximately 40% vs. >80%). Unlike classic SM, which is characterized by aberrant MC morphology, such as spindle shapes and hypogranulation, and associated with the *KIT* D816V mutation [1], a subgroup of patients (<10% of all SM) present with well-differentiated SM (WDSM), characterized by a mature MC morphology (round, fully granulated) and a reduced frequency of *KIT* D816V mutations compared with other forms of SM (29% vs. 93%) [18,19]. Chronic MCL is less aggressive than acute MCL, and chronic MCL is associated with morphologically mature MCs and is less frequently associated with KIT mutations than acute MCL [5]. Collectively, KIT mutations alone cannot explain the full clinical spectrum of SM. Moreover, the *KIT* D816V mutation burden does not correlate with clinical manifestations of ISM [20].

Although KIT mutations, particularly *KIT* D816V, have been considered to serve as key mutations in mastocytosis, an increasing body of data has indicated that other events (e.g., tropomyosin-related kinase [TRK]) may play important roles in the pathogenesis of mastocytosis (Table 1). New data suggested that *KIT* D816V is a late event in the pathogenesis of SM (Table 1) [8]. Moreover, animal studies have suggested that cooperating events are required for the effects of the *KIT* D816V mutation during the induction of SM [21,22]. Interestingly, recent data have cumulatively suggested that the effects of constitutive KIT signaling are dependent on the developmental stage of the cell, targeted by the gain-of-function mutation. In mastocytosis patients, mutations that targeting undifferentiated progenitors have been associated with result in multi-lineage involvement, and resulting in the expression of a severe systemic disease phenotype; in contrast, mutations that target committed MC progenitors or mature MCs alone lead to milder forms of the disease [10].

Determining whether mutated *KIT* is essential for the survival of malignant MCs remains an important goal. The identification of early cooperating events for KIT mutations may improve our understanding of the pathogenesis of SM, leading to more efficient treatments and improved outcomes for SM patients.

### 2.2. TRK (Tropomyosin-Related Kinase)

**Figure 1:**
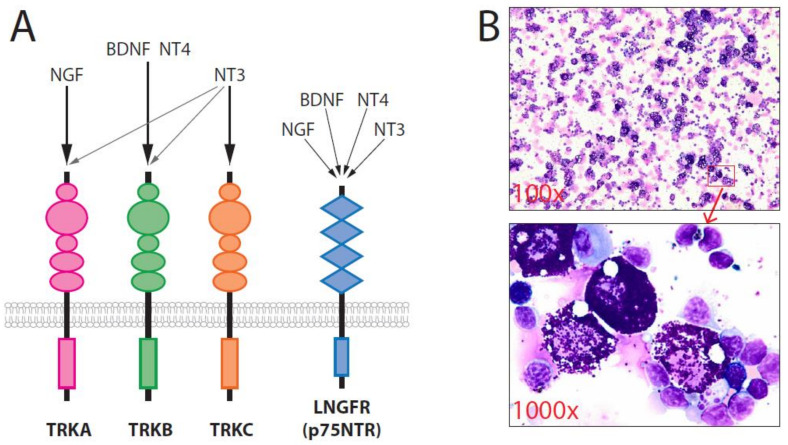
(**A**) The neurotrophin family has four members: NGF, BDNF, NT-3, and NT-4. BDNF, NT-3, and NT-4 can activate TRKB. (**B**) Overexpression of wild-type *TRKB/BDNF* in murine primary hematopoietic stem/progenitor cells induced SM. Cytospins showing strong infiltration of mature MCs in the spleen [37].

The neurotrophins (NTs), which include nerve growth factor (NGF), brain-derived neurotrophic factor (BDNF), NT-3, and NT-4, play major roles in neuronal survival. NTs are unique because they utilize two different classes of receptors: the tropomyosin-related kinases (TRK), including TRKA, TRKB, and TRKC, and the low-affinity NGF receptor (LNGFR = p75NTR) [30,31]. NGF binds most specifically to TRKA, BDNF and NT-4 bind to TRKB, and NT-3 binds to TRKC. NT-3 can also induce the activation of TRKA and TRKB, in addition to TRKC. p75NTR can bind to all four NTs and regulate the affinity of TRK receptors for individual NTs (Figure 1). The promotion of neuronal cell survival by NT requires the activation of TRK, which triggers a Ras-dependent pathway that activates the mitogen-activated protein kinase (MAPK)/extracellular signal-regulated kinase (ERK) and phosphoinositide 3-kinase (PI3K)/protein kinase B (Akt) signaling pathways. TRK family members are expressed by several non-neural cell types and may also play crucial roles in the initiation, progression, and metastasis of many tumors in humans, including breast cancer. Aberrant *TRKB* signaling appears to be sufficient for the induction of tumorigenesis and metastasis [32], cell proliferation, and neoangiogenesis. In addition, some data have indicated the relevance of TRK receptors as prognostic factors [30]. Some TRK inhibitors are currently being tested in clinical trials, and entrectinib and Larotrectinib (LOXO-101) have been approved for use in patients with *TRK* fusion-positiv solid tumors, demonstrating that some human cancers are TRK dependent and that *TRKs* may be good targets for molecular therapy [33,34,35]. Notably, mutated *TRK*/*TRK* fusion is only found in 0.1–0.3% of human cancers [36]. 

The roles played by TRK receptors and their respective ligands during normal and malignant hematopoiesis are not yet well-understood. However, we and others have obtained evidence suggesting an important role for NT signaling in leukemogenesis and mastocytosis [30,38,39,40,41]. Moreover, TRK signaling has been suggested to be involved in other hematological malignancies, such as multiple myeloma. NTs have been known for many years to promote MC chemotaxis, maturation, and survival [40,42,43,44]. For instance, NGF can prevent apoptosis in cord-blood-derived human cultured MCs, acting synergistically with SCF [45]. NGF upregulates the expression of human MC factors, such as tryptase, and immature human MCs can be induced to assume a more mature phenotype in response to NGF treatment in vitro. NGF might have different biological effects at different stages of MC development [42]. Tryptase derived from human MCs can cleave pro-NGF, and mature NGF can be generated in response to tryptase activity [46], which may fundamentally modify the activities of pro-NGF/NGF [46]. Of note, MCs express the mRNA for all NTs and release active NGF, NT-3 (which can activate all TRKs), and NT-4 (which can activate TRKB) [47]. However, the role played by NT signaling in mastocytosis development remains largely unknown. An early report showed that NGF might be a major co-factor for the release of MC-mediators in SM without *KIT* D816V mutation [48]. Recently, Peng et al. demonstrated that patients with mastocytosis had enhanced NT levels and the elevated expression of TRKs on MCs in the skin and gut, and NGF increased the migration of KIT^+^ cells from the blood via TRKA, suggesting that TRK signaling might contribute to mastocytosis pathogenesis through autocrine and paracrine loops [40]. However, the precise contributions of *TRK*s to mastocytosis development and the underlying mechanisms remain largely unclear.

A clear phenotype-genotype correlation has been reported for *TRKA* and *TRKB*. Recently, we demonstrated for the first time that the activation of TRKB by BDNF and the activation of TRKA by NGF in murine hematopoietic stem cells (HSCs) and progenitor cells could efficiently induce a disease with striking similarities to human SM in vivo [29,37]. In 64 mice transplanted with retrovirally gene-modified primary HSC and progenitor cells, we observed SM development in the *TRKA/NGF* (3/7 = 43%) and *TRKB/BDNF* (12/17 = 71%) groups. SM primarily affected the spleen (Figure 1), liver, and bone marrow with multifocal, compact MC infiltrates. MCs mainly exhibited features of mature hypergranular MCs expressing transgene (e.g., *TRKB/BDNF*), *c-Kit, tryptase*, high-affinity receptors for IgE (*FcεRI*) and CD25. Most SM animals followed an indolent disease course. In >100 of our historical control mice transplanted in similar settings with different genes, including *dTRKA*, *dLNGFR*, *FLT3* mutants, *tCD34*, and *SV40* LT, no other mice developed SM [37]. These data strongly suggested that the activation of TRKA and TRKB by their ligands represents an important step in the promotion of mastocytosis. Our data indicated that both TRKA and TRKB activation were more potent than *KIT* D816V for the induction of SM [37], as the retroviral-mediated expression of *KIT* D816V failed to induce SM in transplanted mice [21]. In our models, the MC disease induced by *TRKA/NGF* and *TRKB/BDNF* was strikingly similar to human SM, particularly WDSM. The criteria for chronic human MCL were fulfilled in some mice. Although not all aspects of the human condition were reproduced in our models, the data indicated that TRKA and TRKB activation might play a critical role in the pathogenesis of SM without *KIT* D816V mutation, particularly in the development of WDSM or chronic MCL. Thus, targeting TRKs in such patients might represent a useful therapeutic strategy. 

We found that the activation of TRKA or TRKB was involved in the development of resistance to KIT inhibitors mast cells with *KIT* mutations [29]. Targeting both TRK and KIT significantly prolonged the survival of mice xenotransplanted with HMC-1 cells compared with targeting KIT alone. The induction of TRKA activation in HMC-1 MCL cells that were resistant to KIT inhibition led to the reactivation of the MAPK/ERK signaling pathway and strong upregulation of early growth response 3 (*EGR3*, 1.269-fold), suggesting an important role for the *MAPK-EGR3* axis in the development of resistance to KIT inhibition. Collectively, these data suggested that TRKA signaling may improve neoplastic MC fitness, which would explain, at least in part, why the results of treatment with KIT inhibitors alone in SM patients have been disappointing in most studies [1]. Because NGF is also expressed by bone marrow stromal cells [30], it might activate TRKA and protect MCs from cell death induced by KIT inhibition.

Taken together, we provided the first direct evidence to support SM development induced by the activation of TRKA or TRKB in HSC and progenitor cells in vivo [29,37]. Our data strongly supported the findings described by Peng et al. and their hypothesis [40], suggesting that TRKs have an important role in mastocytosis pathogenesis and the development of resistance to KIT inhibition.

We found that all TRK receptors were expressed on the surface of LAD2 cells, a commonly used MC line, and on primary malignant MCs derived from SM patients ([29] and own unpublished data). Because TRK expression can be identified in almost all patients with SM [40], together with the data from our group showing the induction of SM by *TRKA* and *TRKB* activation, TRK signaling may represent a disease-initiating molecular lesion. A synergistic anti-apoptotic effect has been observed for NGF/SCF and NT-3/SCF in human MCs [45,49]; thus, determining whether TRK signaling cooperates with KIT mutations in the induction of SM remains an important aspect for understanding mastocytosis development. One interesting question is whether the dysregulation of TRK signaling can modify the SM phenotype elicited by KIT mutations (i.e., promoting ISM vs. more aggressive SM subtypes). It is also important to investigate whether TRK is required for KIT signaling to induce mastocytosis.

### 2.3. mTOR Complexes

Mechanistic/mammalian target of rapamycin (mTOR) serves as a central regulator of cell growth and metabolism, forming two distinct complexes, mTORC1 and mTORC2, both of which phosphorylate multiple substrates [50,51]. The fine-tuning activity of mTOR complexes plays an important role in both the maintenance of HSCs and the suppression of leukemogenesis. The aberrant activation of the PI3K/mTOR pathway is a common feature of many cancers, including SM, and represents an attractive target for therapy [52]. Early studies have demonstrated that treatment with the mTOR inhibitor rapamycin depleted leukemic stem cells (LSCs) and restored the functions of normal HSCs [53]. Recent data showed that the depletion of mTORC1 activity failed to eliminate AML stem cells [54]. Interestingly, both *Raptor* and *Rictor* are important for B cell development, but the deletion of *Raptor* or *Rictor* does not alter progenitor cell survival or proliferation [54]. Recent data suggested that targeting Rictor/mTORC2 may be advantageous over the selective targeting of mTORC1 and may represent an attractive alternative approach for the eradication of tumor stem cells while sparing normal stem cell function [55,56,57]. Thus far, many inhibitors targeting the mTOR kinase have been developed; however, the therapeutic effects for SM have not been as robust as expected [50,56,58]. Moreover, very few reports have addressed the role played by the mTOR complexes in SM [52,59,60]. Although mTORC1 may contribute to MC survival, mTORC2 was only found to be critical for the homeostasis of neoplastic and dividing immature MCs [52]. However, no in vivo animal study has addressed the roles played by mTORC1 and mTORC2 in the pathogenesis of SM. Advances in our understanding of how mTOR signaling is involved in the homeostasis of normal HSCs and LSCs may lead to novel therapeutic approaches that may improve the clinical outcomes of patients with SM.

### 2.4. MAPK/ERK

ERK1 and ERK2 play key roles in cell survival, proliferation, cell adhesion, migration, and differentiation in many tissues [61]. ERK1/2 are required for the maintenance of HSCs and immature progenitor cells. The genetic disruption of ERK1/2 protected against the development of myeloproliferative neoplasms (MPN) in a mouse model [62]. ERK signaling appears to be important for the functions of MCs and might be involved in mastocytosis development [43,63]. Interestingly, *dTRKA* did not activate MAPK/ERK signaling, whereas the strong activation of ERK was observed following the TRKA activation by NGF. None of >20 C57BL/6J mice transplanted with *dTRKA*-modified primary Lin-cells developed SM, in contrast with the development of SM in 43% of mice transplanted with *TRKA/NGF*, suggesting that ERK may be important for mastocytosis development. Because the role of ERK1 and ERK2 in MCs and SM development has not been well investigated, the investigation of the roles played by ERK1 and ERK2 in SM development mediated by TRK and KIT signaling should be explored to determine whether ERK1, ERK2, or both isoforms are required for SM development.

### 2.5. Other Alterations/Mutations

More recently, new mutations have been identified in mastocytoses, such as *TET2*, *ASLX1*, *SRSF2*, *JAK2 V617F*, *Nras,* and *ETNK1* (ethanolamine kinase 1) [3,16,64,65]. Interestingly, one study suggested that the overall survival was adversely affected by mutations in *SRSF2*, *ASXL1*, and *RUNX1* [64]. In addition, some signaling pathways have been suggested to be important for the pathogenesis of SM, such as signal transducer and activator of transcription 5 (STAT5) and AKT [43,63]. However, these newly identified mutations and signaling pathways are not specific to mastocytosis [66], and the pathogenetic roles, prognostic, and therapeutic impact of the majority of these mutations and pathways remain to be determined [3]. A recently published study demonstrated that co-expression of *kitD814V* mutation (homolog to human *KIT* D816V) and loss of *TET2* led to a more aggressive phenotype in the skin and the digestive tract than KIT D816 V alone [67]. However, increased mast cell accumulation in other organ systems was not observed, and the co-existence of both mutations was insufficient to confer IL-3 independence to BMMCs (mast cells from bone marrow progenitors), suggesting that additional alterations are still required. Loss-of-heterozygosity of *SETD2* was detected in 27/57 (47%) SM patients [68], suggesting a role of SETD2 deficiency in the pathogenesis of SM. Moreover, mutations in the *TP53* gene are not common in SM but might cooperate with *KIT* D816V to initiate SM [69]. Identifying these genetic and /epigenetic alterations and understanding their interactions and the molecular mechanisms involved in mastocytosis is of the utmost importance for developing rationally targeted therapies.

### 2.6. Cytogenetic Abnormalities

So far, there are only a few reports of cytogenetic analyses in SM patients. Interestingly, cytogenetic abnormalities are highly restricted to patients with advanced SM in two recent publications [17,70], suggesting that they are unlikely to be primary events in the pathogenesis of SM but rather that these abnormalities promote progression to advanced SM.

## 3. Cell Origins of SM

It is well known that MCs are derived from HSCs. The experiments from Gall`s group strongly suggest that adult MC progenitors are derived directly from multipotential progenitors [71]. In contrast to transplantation with primary HSCs and progenitor cells, C3H/HeJ mice transplanted with *TRKB*- and *BDNF*-modified 32D cells (murine myeloid progenitors) only developed AML, with no signs of increased numbers of mastocyte [37]. The finding that mastocytosis is induced only when *TRKB* is activated in HSCs and progenitor cells strongly support the accepted view that MCs are derived from HSCs. There are increasing reports of the co-occurrence of SM with hematological malignancies. Nearly all patients with *KIT* D816V-mutated myelodysplastic syndromes (MDS), MPN, or overlapping MDS/MPN exhibited concurrent SM, whereas 44% of patients with AML and *KIT* D816V showed evidence of SM at some point in their disease course [72]. This suggests that a common cell origin for SM and other hematological malignancies might exist. In a single-cell analysis study, Grootens et al. [73] demonstrated that the *KIT* D816V mutation in SM can be found in cells throughout the hematopoietic landscape, supporting the view a malignant clone may arise in the HSC compartment. Whether other hematological progenitors can be transformed and initiate SM remains to be determined.

## 4. Impacts on the Treatment of SM

More efficient treatment strategies are required to improve the outcomes of patients with SM [74]. Avapritinib is a more potent KIT inhibitor than midostaurin and appears to induce complete responses in more patients [13]. Developing more potent KIT inhibitors for the treatment of SM remains an important goal. The potent new inhibitor ripretinib (DCC-2618) broadly inhibits primary and drug-resistant KIT/PDGFRA mutants [75] and is currently being tested in clinical trials. In 2020 ripretinib was first approved in the USA for the treatment of adults with advanced gastrointestinal stromal tumors (GIST) who had already undergone prior treatments with ≥3 kinase inhibitors. Because the KIT D816V mutation alone cannot induce SM in a transplantation model, however, it is obvious that targeting KIT alone will not control the disease in the long term. Therefore, there is an urgent need to develop novel combined therapies for use in SM patients. A clinical trial examining the combination of KIT inhibition and chemotherapy could provide insightful new data. Other combined therapies including KIT and TRK inhibition [29], KIT and proteasome inhibition [68], and those including donor lymphocyte infusion [76] might be efficient SM treatment strategies.

In addition, it remains to be determined whether chimeric antigen receptor (CAR)-T cell therapies are also effective for the treatment of SM. CARs are unique receptors that are generated to target a specific tumor antigen to functionally reprogram T lymphocytes. CAR-T cell therapy has yielded impressive effects in patients with B-lineage acute lymphoblastic leukemia [77], but to date, there have been no reports of the development of CAR-T cell therapy for SM. Because CD123 is aberrantly expressed on neoplastic MCs [78], and CD123 CAR-T cells are now being intensively tested for treatment of AML in several trials [79], testing CD123 CAR-T cells might be a first step in the development of CAR-T cell therapy for SM. 

## 5. Conclusions

SM is a rare hematological malignancy with complex diagnostic and clinical classifications [5,80]. SM research is lagging behind research investigating other common hematological malignancies such as AML and chronic myeloid leukemia, and fewer scientists and physicians are working in the field of SM; thus it is important to strengthen our collaborations to improve understanding of its pathogenesis. A better understanding of the mechanisms underlying the development of SM may yield improved molecular therapies for its treatment. Patient-specific *KIT* D816V induced pluripotent stem cells (so-called iPSCs) from patients with ASM and MCL represent a patient-specific SM disease model for mechanistic and drug discovery studies. The angiokinase inhibitor nintedanib which targets VEGFR, PDGFR, and FGFR is approved for the treatment of non-small-cell lung cancer, idiopathic pulmonary fibrosis, and other lung diseases by the FDA and EMA was identified as a novel KIT D816V inhibitor using this model [81]. These results suggest nintedanib as a new drug candidate for targeted therapy in patients with advanced SM. Different prognostic scoring systems and genetic biomarkers have been reported [82,83,84,85,86]. These can be used to predict survival outcomes and guide therapy decisions. With novel targeted therapies entering clinical trials, there is now hope for SM patients, particularly those with advanced SM who frequently have impaired quality of life or a poor prognosis. Interestingly, bone marrow MCs lack angiotensin-converting enzyme 2 receptors, and concurrent coronavirus disease 2019 (COVID-19) in SM patients reportedly does not impact MC activation symptoms [87]. Moreover, patients with CM and SM with MC activation symptoms and anaphylaxis reportedly tolerate mRNA COVID-19 vaccination well [88]. We hope that the quality of life of SM patients will be further improved [89].

## Figures and Tables

**Table 1:** Data supporting the important roles of events other than *KIT* mutations in mastocytosis pathogenesis.

	Data and References
1	Although both childhood- and adult-onset mastocytosis are associated with activating *KIT* mutations, the clinical presentation and outcomes of these two conditions differ (a skin-limited disease that spontaneously regresses with age vs. persistent, multi-organ involvement, often with a concurrent non-MC hematologic neoplasm) [3,23]. *KIT* mutations alone cannot explain the full clinical spectrum of SM.
2	KIT D816V does not activate mast cells to release proinflammatory mediators [24].
3	*KIT* D816V mutation appears to be a late event in the pathogenesis of mastocytosis [8].
4	*KIT* D816V is a weak oncogene. For example, BaF3 cells with conditional expression of *KIT* D816V did not form tumors in nude mice [25].
5	*KIT* D816V is thought to promote MC differentiation and maturation rather than MC proliferation [5].
6	The retroviral-mediated expression of *KIT* D816V failed to induce SM in transplanted animals [21]. Only 29% of transgenic mice expressing human *KIT* D816V developed mastocytosis at an old age (>12 months) [22]. In addition, 50% of transgenic zebrafish expressing *KIT* D616V demonstrated a myeloproliferative disease phenotype, including features of ASM [26]. Although mastocytosis was observed in all transgenic mice expressing murine *KitD814V* (homolog of the human D816V mutant) in another model, the disease occurred significantly later and progressed slower when *KitD814V* was expressed in mature MCs [27]. Mastocytosis was not observed at all in transgenic mice expressing *KitD814V* in an earlier study [28].
7	Targeting *KIT* in SM may produce a substantial reduction in MC burden in some patients, however, treatment with KIT inhibitors alone, thus far, has been disappointing in most patients with mastocytosis [1], likely due to the development of resistance to kinase inhibitors [4,29]. A recently published study demonstrated a therapeutic benefit in patients with advanced SM associated with the use of midostaurin (PKC412), a multiple kinase inhibitor that inhibits KIT [11]; however, overall survival at 3 years was only 46%. Few, if any, patients achieved complete remission.
